# Effect of maternal diet on the frequency of micronuclei in pregnant women and newborns: A protocol for systematic review and meta-analysis

**DOI:** 10.1371/journal.pone.0300714

**Published:** 2024-03-25

**Authors:** Anny Cristine de Araújo, Marília Cristina Santos de Medeiros, Priscila Kelly da Silva Bezerra do Nascimento, Ricardo Ney Cobucci, Raul Hernandes Bortolin, Adriana Augusto de Rezende

**Affiliations:** 1 Nutrition Postgraduate Program, Center for Health Sciences, Federal University of Rio Grande Do Norte, Natal, Brazil; 2 Health Sciences Postgraduate Program, Center for Health Sciences, Federal University of Rio Grande do Norte, Natal, Brazil; 3 Sciences Applied to Women’s Health Postgraduate Program, Center for Health Sciences, Maternidade Escola Januário Cicco (MECJ/EBSERH), Federal University of Rio Grande do Norte, Natal, Brazil; 4 Biotechnology Graduate Program, Potiguar University, UnP, Natal, Brazil; 5 Boston Children´s Hospital, Harvard Medical School, Boston, MA, United States of America; 6 Department of Clinical and Toxicological Analyses, Federal University of Rio Grande do Norte, Natal, Brazil; Wroclaw University of Environmental and Life Sciences: Uniwersytet Przyrodniczy we Wroclawiu, POLAND

## Abstract

**Background:**

The effects of diet on maternal and child genetic levels have been previously reported. Diet-associated DNA damage, such as the presence of micronuclei (MN), may be related to an increased risk of developing chronic diseases, such as cancer. Such damage is particularly concerning during pregnancy as it can affect the newborn.

**Aim:**

This review will aim to summarize the primary evidence of the impact of diet during pregnancy on micronucleus frequency in the maternal-newborn population.

**Methods:**

This protocol was developed based on the Preferred Reporting Items guidelines for Systematic Reviews and Meta-analyses Protocol. The review was registered with the International Register of Prospective Systematic Reviews on February 17, 2022 (registration number: CRD42022302401). We will use PubMed, Embase, Web of Science, Scopus, Science direct, and Google databases to search for observational studies. This review will include studies that investigate the diet consumed by pregnant women and its effect on the frequency of MN in mothers and newborns without any time or language limitations. For data extraction, researchers will independently review the full text and collect information that characterizes the study and its findings. We will analyze the results by calculating the odds ratio for each type of diet evaluated, accompanied by a 95% confidence interval. We will perform a quantitative synthesis of homogeneous studies to perform a meta-analysis. Micronucleus frequency quantifies the effect and will be presented as the mean and standard deviation or median and interquartile range.

**Expected results:**

This review will aim to identify which dietary patterns during pregnancy may be associated with an increase in the frequency of MN in mothers and their newborns. Understanding the impact of diet on the frequency of MN is essential to deepen studies and to propose strategies that aim to protect the health of the public through food.

## Introduction

The systemic and genetic influence of an inadequate diet on maternal and child health has been a cause of concern and has led to the development of new investigations [1, 2]. Observational and cohort studies that aimed to evaluate the effect of dietary factors on genetic stability used micronuclei (MN) in their assessments, as it is already well established as a cancer biomarker [3]. MN can be measured using the micronucleus cytome assay in lymphocytes (cytokinesis-blocked micronucleus cytome assay [CBMN]) or oral mucosa (buccal micronucleus cytome [BMCyt]) [4]. MN formation occurs in a situation of poor cell division, generating chromosomal breakage or loss, which may originate from natural or induced causes [5].

Some factors are involved in the formation of MN in pregnant women, such as the absence of vitamins and minerals such as vitamin B6, vitamin B12, and folate, which are cofactors for enzymes and essential components of the DNA repair system [5–7]. Therefore, the researchers have sought to investigate the effect of food consumption, dietary interventions and micronutrient and phytochemical supplements to protect against DNA damage [8–11]. However, sometimes its results can be controversial [1, 2, 12].

Currently, a series of systematic reviews have summarized the results of the effect of nutritional factors on the frequency of MN in adults [13, 14], but until now, there is no knowledge that other reviews of the same type have described these results for the maternal and child population. Therefore, it is relevant to list the primary evidence for this audience. For this purpose, this systematic review protocol aims to describe the steps that will be necessary to summarize the results on the effect of maternal diet on the frequency of MN in mothers and their newborns, in order to guarantee transparency in the process of elaboration the systematic review. Furthermore, this protocol plans the use of meta-analysis to statistically integrate the findings to expand understanding of the relationship between nutrition and genomic protection in maternal and child health.

## Methods

The review protocol was prepared according to the Preferred Reporting Items for Systematic Reviews and Meta-analyses protocol (PRISMA-P) guidelines (S1 File) [15]. The protocol was registered in the International Prospective Register of Systematic Reviews (PROSPERO) under the number CRD42022302401. The systematic review will follow the guidelines of the PRISMA [16].

### Ethics

This review will use secondary data published and available online in electronic databases, so ethical approval and participant consent are not required. Updates will be made to this protocol on the registered platform when applicable.

### Inclusion criteria

To compose this review, the inclusion criteria will be observational studies (cross-sectional, cohort, and case-control studies) published until August 2023. Based on the PECOS structure (Table 1), studies will be included to investigate the diet consumed by pregnant women (without chronological and gestational age restriction) using a food consumption frequency questionnaire (FFQ) and the frequency of MN in these women and their newborns will be evaluated. No restrictions were applied on the year of publication and language.

**Table 1:** PECOS strategy.

Definition	Abbreviation	Elements
**Population**	P	Pregnant women
**Exposure**	E	Inadequate maternal diet
**Control**	C	Proper maternal diet.
**Outcome**	O	Frequency of micronuclei evaluated in cells of the oral mucosa, peripheral blood of pregnant women, and/or umbilical cord blood of newborns
**Study design**	S	Observational studies (cross-sectional, case-controls, cohorts).

### Exclusion criteria

Studies that evaluated the frequency of MN in pregnant women with comorbidities such as gestational or previous diabetes, obesity, chronic and pregnancy-induced hypertension, and children older than 28 days of life were excluded. Similarly, *in vitro* studies, preprints, clinical trials, and review articles were excluded.

### The PECOS strategy

The questions were prepared based on the PECOS strategy (participants, exposure, comparisons, results, and types of study), as presented in Table 1 [17].

A systematic literature search will be performed to identify prospective and retrospective cohort, cross-sectional, and case-control studies that investigate the effects of dietary patterns on the frequency of MN in pregnant women and their newborns. The included studies should describe the impact of inadequate diet on pregnant women and their newborns using the BMCyt or CBMN assay. These tests assess the genotoxicity of environmental factors and quantify biomarkers associated with nuclear damage, cancer development, and other chronic diseases [4, 18]. Participants in this research will be healthy pregnant women, without age limitations, who have reported their food consumption and have consented to the analysis of MN through a biological sample (oral mucosa or blood).

FFQ is a food consumption assessment tool used in population studies. This method is recommended because it is sensitive [19] for identifying eating habits and has a good cost-effectiveness ratio [20]. The systematic review intends to capture studies that use the FFQ to collect food consumption data, as it is a method widely applied to the study of the diet of pregnant or lactating women and its association with micronucleus, diseases and other situations in public health [21, 22].

An adequate maternal diet is one in which food that provides macro and micronutrients is sufficient to provide a healthy maternal nutritional status and adequate fetal growth. The Institute of Medicine (IOM) recommends specific nutrient intake amounts for the gestational period in the Dietary Reference Intakes (DRI) [23]. Furthermore, the DRIs are a set of recommendations that can identify possible dietary inadequacies of specific nutrients. Likewise, the European Food Safety Authority [24] recommends a specific nutrient intake for pregnant women. This systematic review will use the DRI [23] parameters for American studies and EFSA [24] for European studies.

In addition to the assessment of the consumption of specific nutrients, the consumption of food groups will be evaluated using the recommendation of the World Cancer Research Fund [25], whenever this is possible. Thus, in a probable assessment of the consumption of food groups, the intake described in Table 2 will be considered an “adequate diet.”

**Table 2:** Intake recommendation for each food group according to the World Cancer Research Fund.

*Group*	*Consumption recommendations for an adequate diet*
Foods of plant origin (fruits, vegetables, greens, beans, whole grains, Seeds, and nuts)	≥ 400g/day or five servings daily
Red meat	350 - 500g/week or 3 servings per week
Processed meats	Zero
Ultra-processed foods	Zero

Consumption in disagreement with the recommendations described above will be considered an “inadequate diet”.

Based on the FFQ, it will be possible to assess food consumption based on three aspects: food groups, macronutrients, and micronutrients. This review will address four groups of maternal feeding: consumption of plant-based foods, red meat, processed meats, and ultra-processed foods. Consumption values in grams will be obtained for each food group, or nutrient analyzed.

The primary outcome will be micronucleus frequency in pregnant women and newborns. To date, no other outcomes will be considered in this review.

### Search strategy

The search algorithm combines terms registered on the Medical Subject Headings platform and is most commonly used in scientific articles with Boolean operators. Table 3 shows an example of the search strategy that will be applied to PubMed.

**Table 3:** Search strategy for PubMed.

Search terms	
1	Pregnancy
2	Gestation
3	Pregnant women
4	Pregnant
5	Infant
6	Newborns
7	Newborns Infants
8	Neonate
9	Mother-child relations
10	OR / 1–10
11	Maternal exposure
12	Diet
13	Maternal diet
14	Maternal nutrition
15	Nutrient
16	Macronutrients
17	Micronutrients
18	OR / 11–18
17	Micronucleus tests
18	Micronuclei, Chromosome-Defective
19	Micronucleus frequencies
20	DNA damage
21	OR / 17–20
22	10 AND 18 AND 21

A search will be carried out in the following databases: PubMed, Embase, Web of Science, Scopus, Science direct, without time or language restrictions (S2 File). A filter restricts the results of studies conducted on humans. The search for gray literature will be conducted on Google Scholar and OpenGrey. All search results will be organized by a reference manager, where duplicate articles will have their copies deleted. A manual search of the records will be performed in the references of the articles included in the review. Three authors independently selected and evaluated the studies (ACA, MCSM, and PKSBM) for eligibility.

### Data collection and analysis

The selection and screening of studies will be performed independently by three reviewers (ACA, MCSM, and PKSBM), and titles and abstracts will be analyzed based on the eligibility criteria. This step will be performed using Mendeley® software.

**Fig 1:**
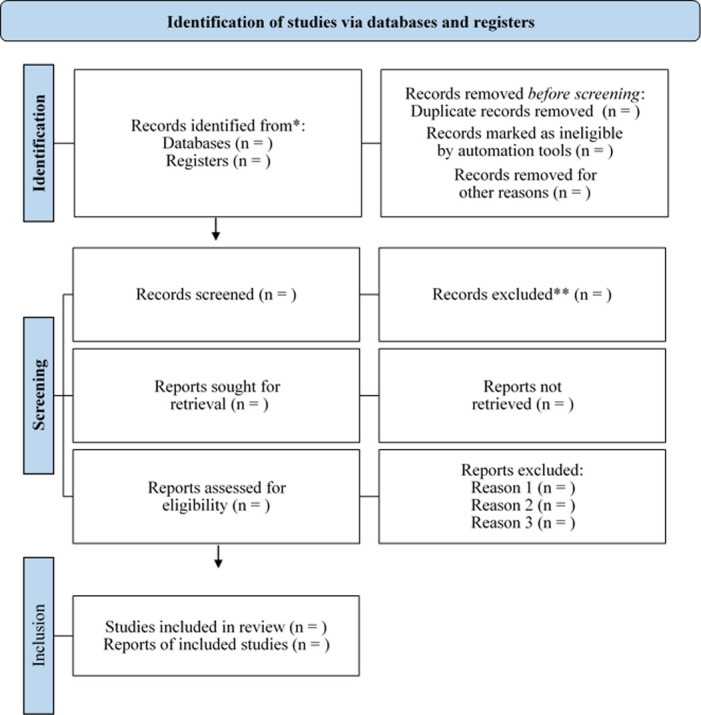
PRISMA diagram for selection of studies included in the systematic review.

The same reviewers will independently access the full text of the selected studies. Only studies that met the eligibility criteria will be included in the review after a full reading by the three reviewers. In the event of disagreement, the opinion of the fourth reviewer (AAR) will be required. During the review stages, the reasons for excluding studies will be recorded. The study selection process is illustrated in Fig 1.

### Data extraction

The same researchers (ACA, MCSM, PKSBM) will independently review the full text and extract the following data: first author, year of publication, year of study, country of research, total number of participants, type of diet consumed, methodology used (CBMN or BMCyt), statistical analysis, and main results. The data will be entered in a specific form in an Excel® spreadsheet created by the reviewers. The data will be compared, and if there is no consensus, the opinion of a fourth reviewer (AAR) will be required.

### Addressing missing data

Reviewers (ACA, MCSM, PKSBM) will contact the authors or co-authors of the article in the case of studies with incomplete, suppressed, or missing data. Contact will be made via email. If there is no response or the availability of information is unfeasible, the data will be excluded from the analysis and addressed in the discussion.

### Quality assessment

The selected studies will be evaluated for methodological quality according to the modified Newcastle-Ottawa Scale (NOS) [26]. The scale application consists of assigning one point (one star) to the items, except for the item “Comparability,” in which the score ranges from zero to two stars. The quality rating will consider the final score: high-quality studies will have a score greater than or equal to six stars; moderate quality, four or five stars; and low quality, less than four stars. Three reviewers will independently perform this step (ACA, MCSM, and PKSBM). The data will be compared, and in case of discrepancy, the opinion of a fourth reviewer (AAR) will be required.

### Assessment of heterogeneity

#### Measures of treatment effect

Quantification of MN will be presented as the mean and standard deviation or median and interquartile range. Quantitative analysis of the results will be performed using the mean absolute difference for studies that present the mean values of the frequency with equal measurement units. Moreover, this analysis can be achieved if the studies present the standardized mean difference (SMD) results with a confidence interval in cases of frequency measured with different units.

Heterogeneity between assay results will be assessed using the I² test. A value of up to 50% will indicate high heterogeneity between studies, and in this case, a random-effects model will be used to combine studies to calculate the odds ratios and the 95% confidence interval (CI), while if the heterogeneity is low (I² <50%), a fixed effects model will be used. A P-value of < 0.05 will be considered statistically significant. We used RevMan version 5.3.5 software to perform the meta-analysis.

### Data synthesis

Depending on the number of studies and information extracted from the articles in the review, a meta-regression of the data will be performed to identify the effect of possible confounding biases on the frequency of MN. If there is an insufficient number of included studies that make quality meta-analysis difficult or high heterogeneity, we will present the narrative synthesis.

### Subgroup and sensitivity analysis

A sensitivity analysis will be performed to check for significant changes in the estimated prevalence, and the 95% CI will be evaluated. The objective of this study is to identify the possible sources of heterogeneity. Low quality studies will be excluded.

If appropriate, analyses will be performed with the following subgroups: maternal age, maternal ethnic origin, birth weight, gestational age, and sex. The results will be reported individually if significant differences exist between subgroups (p < 0.05), and a test will be applied to analyze these interactions using RevMan version 5.3.5.

## Discussion

The MN assay is an essential biomonitoring test because MN is a biomarker related to different types of DNA damage routinely present in precancerous cells [5]. In addition, the test allows for the analysis of other nuclear alterations, such as nuclear buds and nucleoplasmic bridges. These alterations have also been shown to be biomarkers of genomic instability. Therefore, as established in the literature, this assay appears to be a plausible tool for studying the effect of environmental factors at the genetic level [3].

MN can be measured in the cells of the nasal mucosa, urinary tract, erythrocytes, and other mammals. However, the most frequent forms are in the cells of the oral mucosa and lymphocytes by blocking cytokinesis using cytoclastin B. The test using exfoliated cells of the oral mucosa may have advantages as it is less invasive [4, 5].

MN research aims to study the frequency of these changes in humans. For example, the International Human Micronucleus Project (HUMN) aims to be the largest records database for the results of MN tests performed on oral mucosa and peripheral blood lymphocytes [27].

It is known that the formation of the micronucleus is multifactorial [6]. Thus, it is influenced by sex, smoking, alcohol consumption, chronic diseases, medical procedures, and other confounding factors [7]. Therefore, studies must present a methodology and statistical analysis to minimize the effects of confounding factors [3, 5]. Extensive population studies have used fitted models and independently assessed confounders to analyze their influence on data variation [2, 9].

In measuring MN frequency, we can study which foods, habits and/or dietary patterns present themselves as possible risk factors for cancer and other chronic diseases development in the population. Recently, a systematic review of randomized clinical trials and prospective studies summarized the main evidence on the effect of consuming rich foods and beverages and/or supplements with micronutrients or phytochemicals on DNA damage. These damages included the frequency of MN in lymphocytes or oral mucosa. According to the results of the study, some nutrients demonstrated protective effects for adult individuals [14]. Also, a review of 21 studies suggested the clinical applicability of the MN test as a predictor of head and neck cancer [28]. Furthermore, previous reviews and current studies point out the relationship between MN and cancer and corroborate this by highlighting diet as another axis connected to this interaction [1, 4, 29, 30]. For example, for adult individuals, it has been reported that high consumption of processed meat and low intake of food sources with antioxidant components have been associated with some types of cancer [31].

However, for the maternal and child population, there is still an attempt to understand what relationship is established between the effect of maternal diet, the frequency of MN and its implications. A study involving 188 mothers and newborns was proposed to evaluate the influence of food consumption on the frequency of MN [1]. The results showed that dietary patterns characterized by a high consumption of red meat or rich in processed foods increased the frequency of MN in women. This type of diet may have genotoxic substances or low vitamin content, and both conditions can lead to chromosomal loss and alterations in cell segregation and division [3, 4]. Other studies have described these processes as factors involved in MN formation [27, 32].

The study by Furness et al. [33] is one of the first investigations that demonstrate that the frequency of MN in Australian pregnant women may be associated with the development of pre-eclampsia. Some studies also indicate that the frequency of maternal MN has implications for the newborn at a genetic level [2, 6, 9].

A robust evaluation of a dataset conducted in 2015 showed a correlation between food consumption during pregnancy and childhood leukemia and other types of cancers [11, 34]. In this sense, a recent meta-analysis [35] evaluated nine studies that quantified MN in children, especially preschool-aged children with chronic diseases. A seven-fold higher frequency of MN was observed compared to that in the control groups, demonstrating the high risk of exposure to environmental factors. This meta-analysis also highlights some results in newborns and uses them to affirm that blood collection through the umbilical cord can be a viable methodological alternative. However, it does not delve into the analysis of external factors, especially diet quality and/or pattern, as we suggest.

Hoping to contribute to elucidating knowledge on this topic, our review will aim to summarize the factors that may have transgenerational effects and reinforce appropriate habits during pregnancy. The design of this review will use secondary data to answer the research question. Thus, our limitations are related to the results of the data analysis of each article, the quality, and the number of studies included. These factors may compromise the reliability of the results.

We hope that the results make it possible to raise a discussion about how the type of maternal diet influences the frequency of MN in pregnant women and their newborns. Knowing the effect of a factor on the frequency of MN is essential for us to propose strategies that aim to protect and promote the health of this and the next generations.

## Supporting information

S1 File PRISMA-P 2015 checklist. (PDF)

S2 File Search strategy in different databases. (PDF)
