# Correction: Effect of maternal diet on the frequency of micronuclei in pregnant women and newborns: A protocol for systematic review and meta-analysis

**DOI:** 10.1371/journal.pone.0308937

**Published:** 2024-08-12

**Authors:** Anny Cristine de Araújo, Marília Cristina Santos de Medeiros, Priscila Kelly da Silva Bezerra do Nascimento, Ricardo Ney Cobucci, Raul Hernandes Bortolin, Adriana Augusto de Rezende

There are errors in the Funding statement. The correct Funding statement is as follows: This study was paid by the Coordenação de Aperfeiçoamento de Pessoal de Ensino Superior—Brasil (CAPES)—Funding Code 001, via the Federal University of Rio Grande do Norte (UFRN). CAPES had no role in study design, data collection and analysis, decision to publish, or preparation of the manuscript.
