# Supplementary material for: Effect of maternal diet on the frequency of micronuclei in pregnant women and newborns: A protocol for systematic review and meta-analysis
Source: PLoS One. 2024 Mar 25;19(3):e0300714. doi: 10.1371/journal.pone.0300714 (PMC10962814; doi:10.1371/journal.pone.0300714)
Supplement: S2 File — (PDF) [file pone.0300714.s002.pdf]

S2 File: Search strategy in different databases

Databases	Search strategy	Results
Pubmed	(Pregnancy [MESH terms] OR Gestation OR Pregnant Woman [MESH terms] OR Woman, Pregnant Women OR Pregnant OR INFANT [MESH] (Infants) Newborn [MESH terms] OR Newborn Infants OR Newborn Infant OR Newborns OR Neonate OR Neonates OR Mother-Child Relations [MESH]) AND (Maternal Exposure [MESH terms] OR Exposure, Maternal OR Maternal Exposures OR Diet [MESH terms] OR Maternal Diet OR Maternal Nutrition OR Nutrients [MESH terms] OR Nutrient OR Macronutrients OR Micronutrients) AND (Micronucleus Tests [MESH terms] OR Micronucleus Test OR Micronuclei, Chromosome-Defective [MESH terms] OR Micronucleus frequencies OR DNA damage [MESH terms])	104
Science Direct	(Pregnancy [MESH terms] OR Newborn [MESH terms] OR Mother-Child Relations [MESH]) AND (Maternal Exposure [MESH terms] OR Diet [MESH terms] OR Maternal Nutrition) AND (Micronucleus Tests [MESH terms] OR Micronuclei frequency OR Micronuclei, Chromosome-Defective [MESH terms])	43
Web of Science	((((((((((((((((((((((((((TS=(Pregnancy [MESH terms])) OR TS=(Gestation)) OR TS=(Pregnant Woman [MESH terms])) OR TS=(Woman, Pregnant Women)) OR TS=(Pregnant)) OR TS=(INFANT [MESH] (Infants))) OR TS=(Newborn [MESH terms])) OR TS=(Newborn Infants)) OR TS=(Newborn Infant)) OR TS=(Newborns)) OR TS=(Neonate)) OR TS=(Neonates)) OR TS=(Mother-Child Relations [MESH])) AND TS=(Maternal Exposure [MESH terms])) OR TS=(Exposure, Maternal)) OR TS=(Maternal Exposures)) OR TS=(Diet [MESH terms])) OR TS=(Maternal Diet)) OR TS=(Maternal Nutrition)) OR TS=(Nutrients [MESH terms])) OR TS=(Nutrient)) OR TS=(Macronutrients)) OR TS=(Micronutrients)) AND TS=(Micronucleus Tests [MESH terms])) OR TS=(Micronucleus Test)) OR TS=(Micronuclei, Chromosome-Defective [MESH terms])) OR TS=(Micronucleus frequencies)) OR TS=(DNA damage [MESH terms])	349
Embase	(pregnancy'/exp OR pregnancy OR 'pregnant woman':ti,ab OR newborn:ti,ab OR 'mother child relation':ti,ab) AND ('maternal exposure':ti,ab OR 'maternal nutrition':ti,ab OR diet:ti,ab OR nutrient:ti,ab) AND 'micronucleus test':ti,ab OR micronucleus:ti,ab)	90
Scopus	(TITLE-ABS-KEY (pregnancy) OR TITLE-ABS-KEY (gestation) OR TITLE-ABS-KEY ("pregnant woman") OR TITLE-ABS-KEY (pregnant) OR TITLE-ABS-KEY (infant) OR TITLE-ABS-KEY (newborn) OR TITLE-ABS-KEY ("newborn infants") OR TITLE-ABS-KEY ("newborn infant") OR TITLE-ABS-KEY (newborns) OR TITLE-ABS-KEY (neonate) OR TITLE-ABS-KEY (neonates) OR TITLE-ABS-KEY ("mother-child relations") AND TITLE-ABS-KEY ("maternal exposure") OR TITLE-ABS-KEY (diet) OR TITLE-ABS-KEY ("maternal diet") OR TITLE-ABS-KEY ("maternal nutrition") OR TITLE-ABS-KEY (nutrients) OR TITLE-ABS-KEY (nutrient) OR TITLE-ABS-KEY (macronutrients) OR TITLE-ABS-KEY (micronutrients) AND TITLE-ABS-KEY ("micronucleus tests") OR TITLE-ABS-KEY ("micronucleus test") OR TITLE-ABS-KEY ("micronuclei, chromosome-defective") OR TITLE-ABS-KEY ("micronucleus frequencies"))	59
Scholar Google	(Pregnancy OR Mother-newborns OR Newborn) AND (Dietary estimates OR Maternal diet) AND (Micronuclei frequency OR Micronucleus frequencies)	3700
